# Normalized Index of Synergy for Evaluating the Coordination of Motor Commands

**DOI:** 10.1371/journal.pone.0140836

**Published:** 2015-10-16

**Authors:** Shunta Togo, Hiroshi Imamizu

**Affiliations:** 1 Cognitive Mechanisms Laboratories, Advanced Telecommunications Research Institute International, Kyoto, Japan; 2 Japan Society for the Promotion of Science, Tokyo, Japan; 3 National Institute of Information and Communications Technology, Osaka, Japan; 4 Department of Psychology, The University of Tokyo, Tokyo, Japan; Tokai University, JAPAN

## Abstract

Humans perform various motor tasks by coordinating the redundant motor elements in their bodies. The coordination of motor outputs is produced by motor commands, as well properties of the musculoskeletal system. The aim of this study was to dissociate the coordination of motor commands from motor outputs. First, we conducted simulation experiments where the total elbow torque was generated by a model of a simple human right and left elbow with redundant muscles. The results demonstrated that muscle tension with signal-dependent noise formed a coordinated structure of trial-to-trial variability of muscle tension. Therefore, the removal of signal-dependent noise effects was required to evaluate the coordination of motor commands. We proposed a method to evaluate the coordination of motor commands, which removed signal-dependent noise from the measured variability of muscle tension. We used uncontrolled manifold analysis to calculate a normalized index of synergy. Simulation experiments confirmed that the proposed method could appropriately represent the coordinated structure of the variability of motor commands. We also conducted experiments in which subjects performed the same task as in the simulation experiments. The normalized index of synergy revealed that the subjects coordinated their motor commands to achieve the task. Finally, the normalized index of synergy was applied to a motor learning task to determine the utility of the proposed method. We hypothesized that a large part of the change in the coordination of motor outputs through learning was because of changes in motor commands. In a motor learning task, subjects tracked a target trajectory of the total torque. The change in the coordination of muscle tension through learning was dominated by that of motor commands, which supported the hypothesis. We conclude that the normalized index of synergy can be used to evaluate the coordination of motor commands independently from the properties of the musculoskeletal system.

## Introduction

Humans perform motor tasks despite many redundancies in our bodies. In general, a performance variable that must be controlled to achieve a task (e.g. hand positioning) is controlled by redundant motor elements (e.g. joint angles). Recently, it has been reported that humans perform various tasks by coordinating redundant motor elements to stabilize the required performance variable [1–4]. This coordination is a flexible strategy in which the constant value of the performance variable is maintained across trials, while some variability of motor elements is allowed. A synergy is as a mechanism that performs task-specific coordination of the trial-to-trial variability of motor elements. Previous studies have evaluated these coordinated structures by examining coordination among motor elements, i.e. motor outputs that are affected by the musculoskeletal system. However, the question of whether an observed synergy is formed by motor commands or properties of the musculoskeletal system remains unsolved. This problem is important in the investigation of the neural mechanisms of synergy formation. To solve this problem, we propose a method that can evaluate the coordination of motor commands by removing the effects of the properties of the musculoskeletal system.

**Fig 1:**
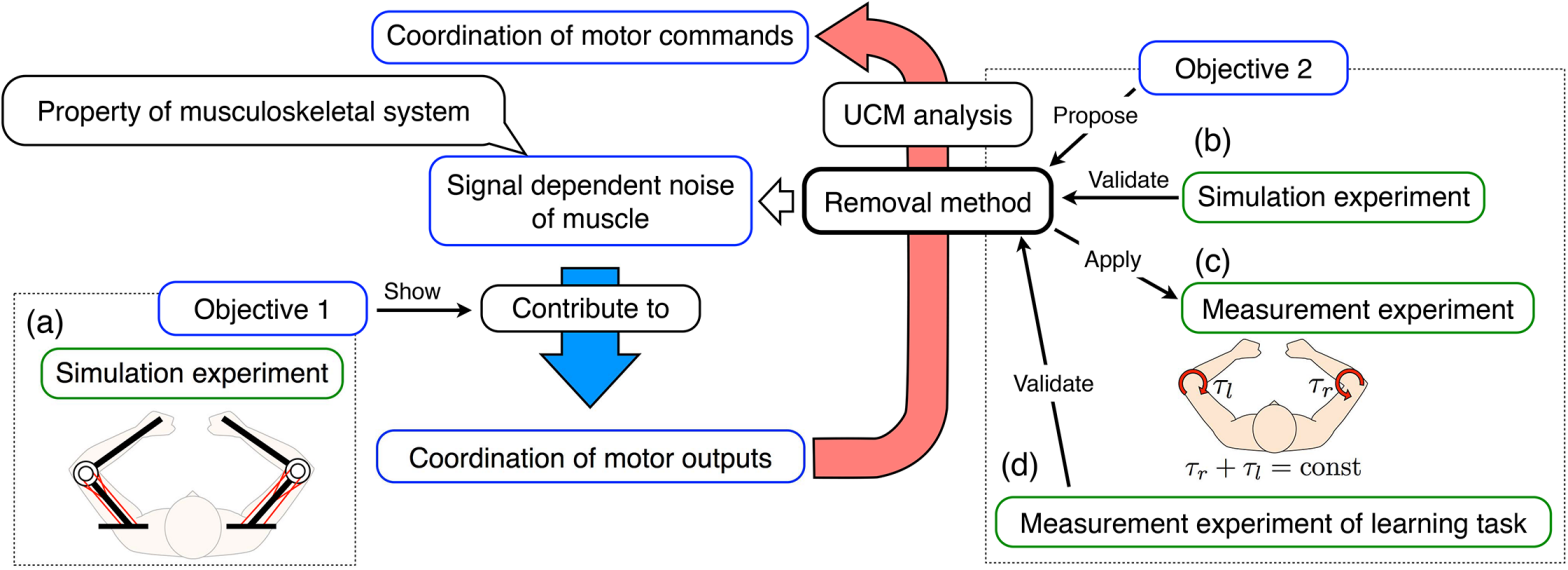
Concept of coordination of motor commands. (a) The simulation experiment shows that signal-dependent noise in the muscle contributes to the coordination of motor outputs. We propose a method that removes the effects of the musculoskeletal system for the assessment of the coordination of motor commands. (b) The simulation experiment validates our proposed method. (c) To show the coordination of motor commands of human subjects, the proposed method is applied to the measurement experiment in which the subjects perform the same task as the simulation experiment. (d) To empirically validate the proposed method, it is applied to the measurement experiment of the learning task.

Because the coordination of movements is defined by the variability of motor outputs, we consider signal-dependent noise (SDN) in the musculoskeletal system that affects the variability of motor outputs [5]. SDN in a muscle is when the standard deviation of the muscle tension linearly increases with the mean muscle tension magnitude [6–7]. The degree of variability of the muscle (the coefficient of variation) is different among body parts [8]. When muscle tensions are considered as motor elements, the difference in the degree of variability of muscle tensions forms a distribution of trial-by-trial variability of the motor elements that expands toward a specific direction in the space of motor elements. Therefore, this difference in degree of variability in each muscle could be the cause of synergies between motor elements. In this study, we first demonstrate using simulation experiments that the variability of muscle tensions due to SDN underlies the formation of synergies (Fig 1A).

Uncontrolled manifold (UCM) analysis has previously been used to quantitatively evaluate coordinated movements based on the variability of motor elements [9]. This analysis uses the UCM that is a subspace of the motor elements constructing the same value of the performance variable, and divides the variance of the motor elements into two components; one is arranged parallel to the UCM (the UCM component), and the other is orthogonal to the UCM (the ORT component). The UCM component does not affect the performance variable, and the ORT component directly affects it. If the UCM component is larger than the ORT component, then the performance variable is controlled through the coordination of the motor elements. The ratio of the UCM and ORT components quantitatively represents a degree of coordination, i.e. a synergy. UCM analysis has revealed synergies in various tasks, from whole body movements [10–12] to multi-finger movements [13–15]. In this study, we use the framework of UCM analysis to evaluate the coordination of motor commands.

To achieve this, we propose a method to remove the effects of SDN. Firstly, the proposed method transforms the observable variability of motor outputs into a signal-independent distribution of motor commands. Then, UCM analysis is applied to calculate a normalized index of synergy that represents the coordination of motor commands. Using simulation experiments, we demonstrate that the normalized index of synergy appropriately represents the coordination of motor commands (Fig 1B). Next, we conduct measurement experiments in which human subjects perform the same task as done in the simulation experiments. The normalized index of synergy is applied to evaluate the coordination of the subjects’ motor commands (Fig 1C). Finally, we evaluate the change in the normalized index of synergy through motor learning to demonstrate the utility of our proposed method (Fig 1D). Because the properties of the musculoskeletal system do not change through short-term learning, we hypothesize that the majority of changes in the coordination of motor outputs through motor learning are dominated by changes in the coordination of motor commands. We test this hypothesis by calculating the ratio of the change of the normalized index of synergy through motor learning to the change of the index of synergy for muscle tension.

## Materials and Methods

### Normalized Index of Synergy

**Fig 2:**
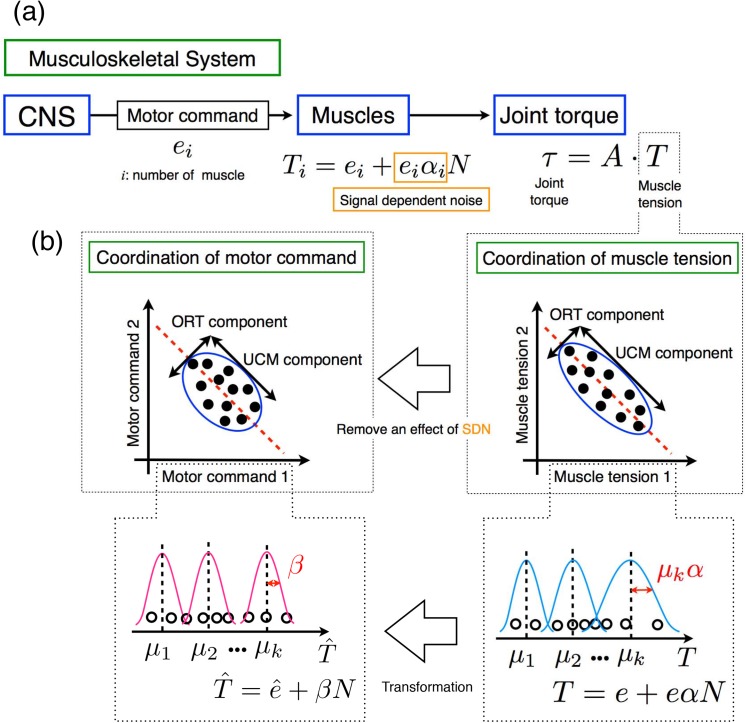
Method for evaluating the coordination of motor commands. (a) The musculoskeletal system considered in this study. The CNS sends motor commands to each muscle. The muscle generates muscle tension with signal-dependent noise. The muscle tensions form the joint torque. (b) Transformation from the coordination of muscle tensions to that of motor commands. Using a coefficient of variation of each muscle, the variability of muscle tensions across trials is transformed so that it is distributed according to a signal-independent mixture of Gaussian distributions. Then, the UCM analysis is applied to quantitatively evaluate the coordination of motor commands.

In this study, we consider a simple musculoskeletal system as shown in Fig 2A. The central nervous system (CNS) sends motor command *e*_*i*_ to each muscle. Because each muscle has SDN properties, the muscle tension of the *i*-th muscle *T*_i_ is defined as follows:

$$T_{i}=e_{i}+e_{i}\alpha_{i}N(0,1),$$ (1)

where *α*_i_ denotes the coefficient of variation of the *i*-th muscle and *N*(0,1) indicates a Gaussian distribution with mean 0 and standard deviation 1. Then, an isometric joint torque τ is produced by a muscle tension *T* as follows:

τ=A⋅T, (2)

where *A* denotes a transformation matrix. We considered an isometric condition; thus, the transformation matrix *A* s constant.

We assumed that the variability of the tension in each muscle differed according to the SDN. In this study, we considered the variability of muscle tension when the effects of SDN on the motor command were removed. To remove the effect of SDN, we transformed the variability that depended on a Gaussian distribution of SDN, as in Eq (1) (Fig 2B right), into a distribution that depended on a Gaussian distribution of signal-independent noise (Fig 2B left) as follows:

$$\hat{T}={\hat{e}}_{i}+\beta N(0,1),$$ (3)

where ${\hat{T}}_{i}$ denotes the motor command; *ê*_1_, an input; and *β*, the standard deviation of the signal-independent Gaussian distribution. We assumed that the value of *β* was invariant across all inputs. Therefore, the difference of degree of variability based on the SDN could be eliminated.

For the transformation, two types of measured data were required. One type was the coefficient of variation α_i_ (in Eq (1)), which is the magnitude of the SDN of each muscle in each subject. The coefficient of variation was determined from the mean and standard deviation of the measured electromyogram (EMG) data when the subject produced isometric hand forces in various directions and at various magnitudes. The other was the trial-to-trial variability of muscle tensions in a redundant motor task that were transformed into the variability of motor commands.

To transform the measured distribution of muscle tensions, the following two steps were required: Firstly, we estimated the number of inputs (*e*_*i*_ in Eq (1)) that generated the measured variability of each muscle tension. The coefficient of variation *α* was determined under the assumption that the same joint torque value is generated by a unique combination of inputs *e*. The variability of muscle tension generated by a single input is distributed according to a single Gaussian distribution (Eq (1)), which is observed in the not-redundant motor task. In the redundant motor task, however, multiple combinations of joint torque are allowed to achieve the task, and thus multiple inputs are used. Therefore, we assumed that the variability of muscle tensions in the redundant task could be generated by a mixture of Gaussian distributions (Fig 2B right). We estimated the mixture of Gaussian distributions depending on the SDN of Eq (1) from the measured variability of muscle tensions by maximum-likelihood estimation of an EM algorithm [16–17]. In general, the EM algorithm estimates three parameters: the mean *μ*, variance *σ* ^*2*^, and the mixing coefficient *π* of the mixture of distributions. In this study, however, we assumed that the variance of the mixture distribution was signal dependent (*σ* ^*2*^ = *α* ^*2*^ *μ* ^*2*^), so we estimated only two parameters: the mean *μ* and the mixing coefficient *π*. The number of the Gaussian mixture distributions *κ* was determined according to a Bayesian information criterion (BIC) [18]. The *k*-th distribution was a Gaussian distribution with mean *μ*_*k*_ and standard deviation *μ*_*k*_ *α*. Secondly, the measured EMG data *T*_*i*_ were transformed so that the mean *μ*_*k*_ was unchanged and the standard deviation was *β* (from Fig 2B right to left) using the following equation:

$${\hat{T}}_{i}=T_{i}+\sum_{k}\gamma_{k}\left(T_{i}-\mu_{k}\right)\left(\frac{\beta }{\alpha_{i}\mu_{k}}-1\right),$$ (4)

where ${\hat{T}}_{i}$ denotes the motor command (Eq (3)); *T*_*i*_, measured muscle tension; and *γ*_*k*_, the responsibility calculated with the EM algorithm. A derivation of Eq (4) is presented in Appendix A. The mean value of *μ*_*k*_ *α*_*i*_ of all muscles was used for the value of *β*. Through the above procedure, the measured distribution of muscle tensions was transformed into that of motor commands.

The UCM analysis was applied to the distribution of motor commands $\hat{T}$ to quantify the coordination of the motor commands. The deviations of $\hat{T}$ from the mean $\bar{\hat{T}}$ are divided into the UCM component *V*_*UCM*_, which does not affect the performance variable, and the ORT component *V*_*ORT*_, which directly affects the performance variable. *V*_*UCM*_ is obtained by projecting the deviation of $\hat{T}$ onto the UCM that is defined by the null space of the transformation matrix *A* in Eq (2) as follows:

$${\hat{T}}_{∥}=\sum_{j=1}^{n-d}\varepsilon_{j}^{T}\cdot \left(\hat{T}-\bar{\hat{T}}\right)\cdot \varepsilon_{j}$$ (5)

$$V_{UCM}=\frac{1}{n-d}{\hat{T}}_{∥}^{T}{\hat{T}}_{∥},$$ (6)

where *ε*_*j*_ denotes a basis vector of the null space of *A*, and *n* and *d* represent the dimensions of the motor elements and the performance variable, respectively. *V*_*ORT*_ is calculated by subtracting *V*_*UCM*_ from the deviation of $\hat{T}$:

$${\hat{T}}_{⊥}=\left(\hat{T}-\bar{\hat{T}}\right)-{\hat{T}}_{∥},$$ (7)

$$V_{ORT}=\frac{1}{d}{\hat{T}}_{⊥}^{T}{\hat{T}}_{⊥}.$$ (8)

The index of synergy is defined by the ratio of *V*_*UCM*_ and *V*_*ORT*_. In the framework of UCM analysis, a high ratio of the UCM component in the variability of all motor elements is interpreted as enhanced coordination of motor elements, because high variability in motor elements that do not affect task achievement indicates a high degree of the coordinated compensation of motor elements used to stabilize the performance variable. A conventional index of synergy is defined as follows:

$$\Delta V=\frac{V_{UCM}-V_{ORT}}{V_{TOT}},$$ (9)

where *V*_*TOT*_ denotes the total variance normalized by the dimension of motor elements. Δ*V* is not distributed according to a Gaussian distribution; hence, we used a Fisher Z-transformed index of synergy [19]:

$$\Delta V*=\log\left(\frac{\Delta V+\frac{n}{d}}{\frac{n}{n-d}-\Delta V}\right).$$ (10)

When the motor elements are not coordinated (*V*_*UCM*_ = *V*_*ORT*_), Δ*V* = 0 and ΔV* = 1.95. Thus, ΔV* > 1.95 specifies the coordination of the motor elements to stabilize the total elbow torque [3]. In this study, we referred to the Fisher Z-transformed index of synergy ΔV* for motor command $\hat{T}$ as the normalized index of synergy.

### Total Elbow Joint Torque Production Task

**Fig 3:**
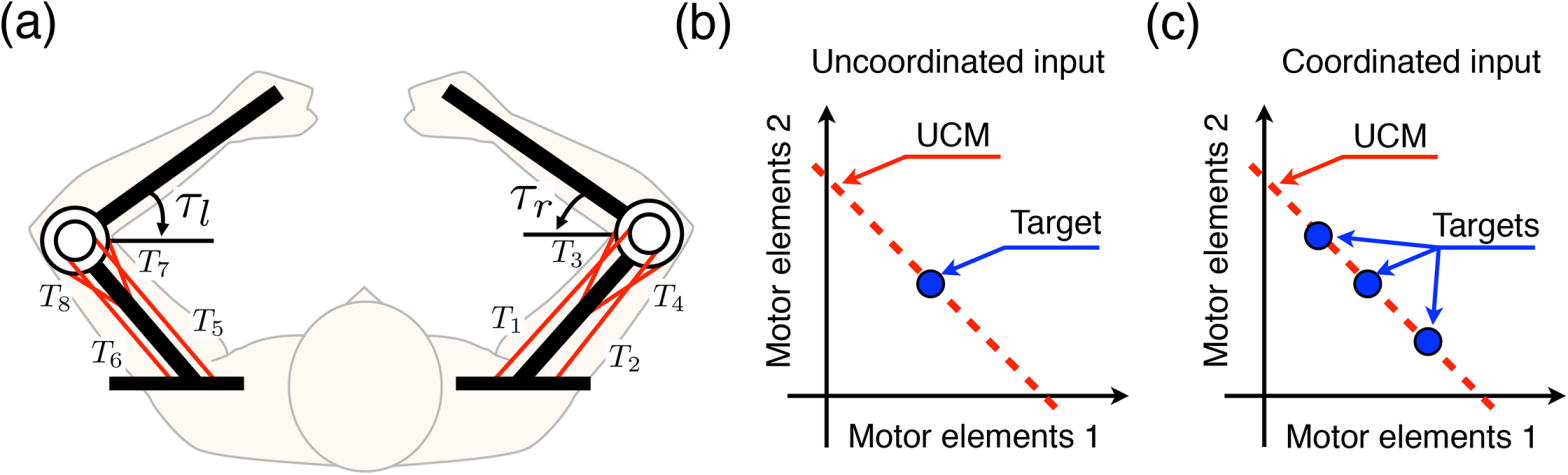
Human elbow model used in the simulation experiments. (a) The two elbow joint, eight muscle model. The four muscle tensions (*T*_1_–*T*_4_) generate the right elbow joint torque *τ*_*r*_, and the others (*T*_5_–*T*_8_) generate the left elbow joint torque *τ*_*l*_. The muscles *T*_3_, *T*_4_, *T*_7_, and *T*_8_ are the monoarticular muscles, and the others are the biarticular muscles. (b) The uncoordinated input corresponds to one point in the UCM. (c) The coordinated input corresponds to three points in the UCM.

In this study, we considered a total elbow joint torque production task, wherein a subject produces isometric total elbow torque using both right and left elbows (Fig 3A). In this task, the performance variable is the sum of the right and left elbow torques, and the motor elements are the eight muscles associated with the flexion and extension of the right and left elbows. In the simulation experiments, we considered a model of a two-elbow joint with eight muscles, as shown in Fig 3A. In the target total torque production task measurement experiments, subjects produced total elbow joint torque toward a target total torque. In the learning task measurement experiments, subjects learned to track a target trajectory consisting of total elbow torque. Details of the experiments are described in the following sections.

### Simulation Experiment

We considered a two-joint, eight-muscle model of human elbow joints as a controlled object (Fig 3A). The muscle tension of the *i*-th muscle *T*_*i*_ was given as Eq (1). The values of the coefficient of variation *e*_*i*_ and the transformation matrix *A* were given as those of a typical subject in the measurement experiments. The first aim of this simulation experiment was to demonstrate that the properties of SDN form the coordination of muscle tensions. We used two kinds of input, arbitrarily determined so that the total torque of the right and left elbow joints was 5 N·m (Table 1). One input was uncoordinated, consisting of a unique combination of inputs *e*_*i*_. The uncoordinated input generated 300 muscle tensions, corresponding to 300 trials, using Eq (2) and a unique *e*_*i*_. The other input was coordinated, consisting of three sets of combinations of inputs *e*_*i*_. The coordinated input also generated 100 muscle tensions per combination of *e*_*i*_, and generated 300 muscle tensions in total. As above, we considered the uncoordinated input as a single input and the coordinated input as multiple inputs to generate the same value of total elbow torque. Therefore, the uncoordinated input corresponded to one point on the UCM (Fig 3B), and the coordinated inputs corresponded to three points on the UCM (Fig 3C). We considered these inputs as motor commands. Applying the UCM analysis, we examined whether the UCM component was larger than the ORT component for the uncoordinated input. The second aim was to demonstrate that our proposed method can appropriately evaluate the coordination of motor commands. The variance in muscle tensions for each input was transformed to that of motor commands by our proposed method, and the UCM analysis was applied to calculate the measured index of synergy (evaluation of the coordination of muscle tensions) and normalized index of synergy (evaluation of the coordination of motor commands). Then, we examined whether the normalized index of synergy for uncoordinated inputs showed a value of no coordination (UCM component = ORT component), and whether the value for coordinated input was higher than that of no coordination. The above sequence consists of a single session of the simulation experiment. Ten sessions were conducted for the statistical evaluation of UCM analysis.

**Table 1:** Inputs for each muscle in the simulation experiments.

	*e*_1_	*e*_2_	*e*_3_	*e*_4_	*e*_5_	*e*_6_	*e*_7_	*e*_8_
Uncoordinated input	0.08	0.01	0.10	0.09	0.08	0.01	0.10	0.29
	0.03	0.02	0.06	0.06	0.15	0.05	0.13	0.38
Coordinated input	0.08	0.01	0.10	0.09	0.08	0.01	0.10	0.29
	0.15	0.04	0.15	0.13	0.03	0.03	0.08	0.21

### Measurement Experiment

#### Subjects

Ten healthy right-handed males participated in the experiments. Their average age was 25.2 (21–28 years). Their dominant hand was assessed using the Edinburgh Handedness Inventory [20]. The experiments were approved by the Ethics Committee at the Advanced Telecommunication Research Institute International, Japan (www.atr.jp). All subjects were briefed on the experimental procedure and gave their written informed consent.

#### Data collection

**Fig 4:**
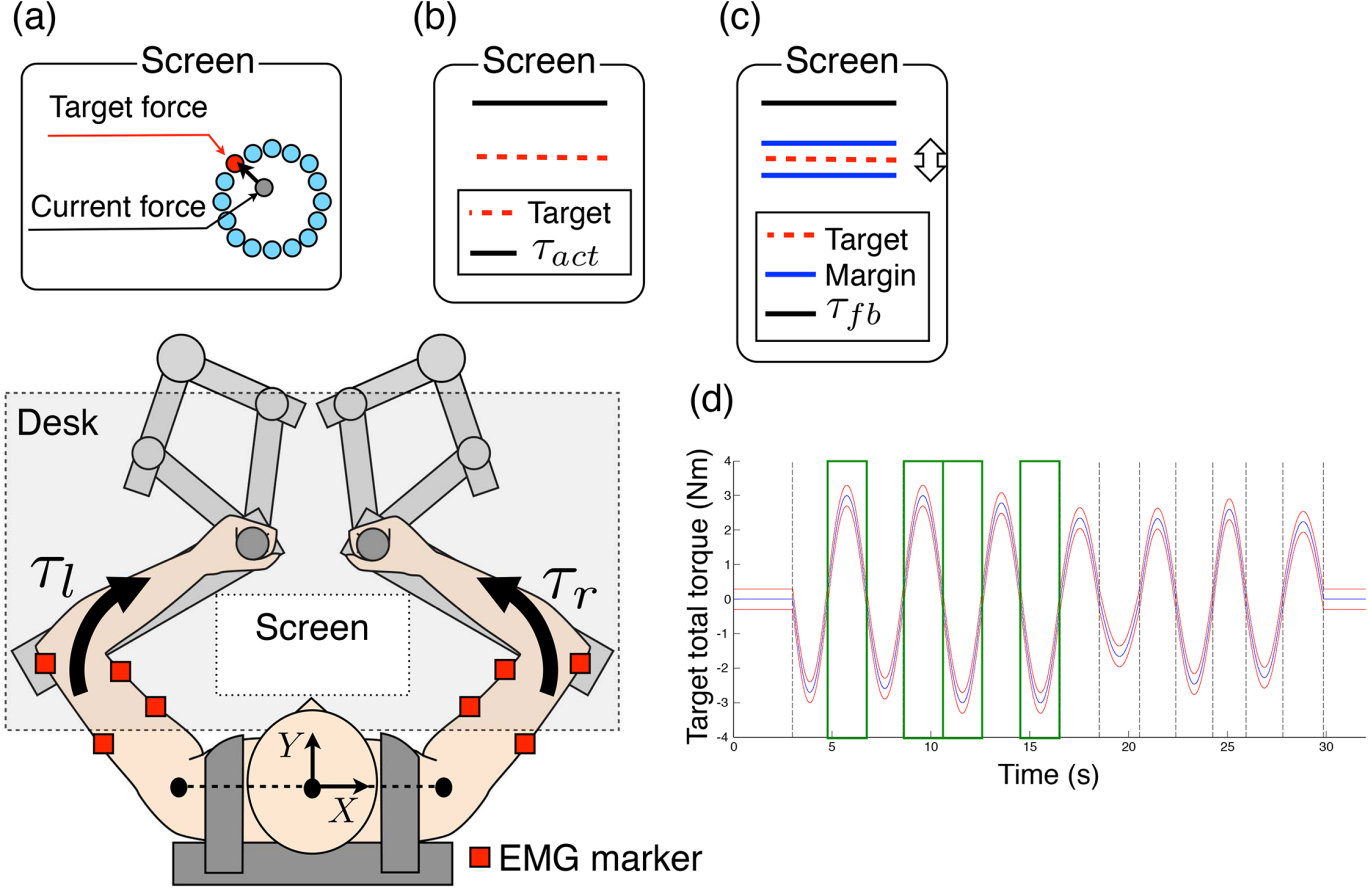
Schematic of the experimental set-up. Subjects were seated on a chair, wearing a seat-belt to fix their trunks, and had force sensors placed in their right and left hands. The red squares denote electrodes used to record the electromyogram data. The screen on the desk showed the information corresponding to the target hand force production task (a), the target total torque production task (b), and the tracking-ability learning task (c). (d) One example of a target trajectory of total elbow torque. The target trajectory consists of 14 half-sine waves. The four half-sine waves in the green boxes are standard half-sine waves (amplitude: ± 3 N·m, period: 4 s), and the others are randomly generated half-sine waves (amplitude: mean ± 3 N·m, standard deviation 1 N·m, period: mean 4 s, standard deviation 0.4 s). The blue line indicates the target trajectory and the red lines indicate the ± 0.3 N·m margin.

We used a twin visuomotor and haptic interface system (TVINS) to record the force of both hands at 2000 Hz (Fig 4). The arms of TVINS were fixed so that they did not move the subject’s hands during measurement. The hand forces were measured by the load cell of TVINS arms, and were transformed into elbow joint torque using the pre-measured length of each subject’s arm. EMG was also recorded from an elbow monoarticular flexor (brachioradialis) and extensor (lateral head of triceps brachii), and a biarticular flexor (biceps brachii) and extensor (long head of triceps brachii) of both hands at 2000 Hz. A projector displayed task instructions on a screen that was placed above the subject’s arm.

#### Procedure

Subjects sat on a chair and wore a seat belt as shown in Fig 4. The hand positions were +45 cm in the *Y*-direction and ±17.5 cm in the *X*-direction from an origin that was defined as the center of the line connecting the centers of gyration of both right and left shoulders. Subjects performed the following three tasks:

1. Target hand force production task: To determine the coefficient of variation *α*_*i*_, subjects produced 16 directional isometric hand forces using their right or left hand, as shown in Fig 4A. The magnitudes of the isometric forces were 5 N, 10 N, and 15 N. Subjects continued to produce the force for 2 s per each directional force. The screen showed the 16 directional targets and the subject’s current hand force. The average value and standard deviation of the EMG of each muscle were used to determine the coefficient of variation *α*_*i*_.
2. Target total torque production task: Subjects produced one-dimensional target total elbow torque, as shown in Fig 4B. They produced elbow flexion torque for 1.5 s so that the sum of the right and left elbow torques was 5 N·m. The target total torque and actual produced total torque *τ*_*act*_ were shown by line positions. Subjects performed 50 trials and were instructed to only produce the target total torque. Balance between right and left elbow torques was not instructed. Measured EMG data were used to evaluate the coordination of the muscle tensions and that of the motor commands.
3. Tracking-ability learning task: Subjects tracked a randomly generated target trajectory of total elbow torque, and learned to improve their tracking performance (Fig 4C). The tracking-ability learning task was designed based on earlier studies using a total finger force production task [21–22]. The target trajectory of total elbow torque in one trial consisted of 14 half-sine waves, as shown in Fig 4D. Four of the half-sine waves were standard half-sine waves (amplitude: ± 3 N·m, Period: 4 s), with 10 of them randomly generated (amplitude: mean ± 3 N·m, standard deviation 1 N·m, period: mean 4 s, standard deviation 0.4 s). The order of these 14 half-sine waves was randomly shuffled trial-by-trial. Therefore, subjects could not learn the unique pattern of the target trajectory, but had to learn the tracking ability itself. The target trajectory was shown by a one-dimensional line with a ± 0.3 N·m margin. To control the task difficulty, the total elbow torque *τ*_*fb*_ that was displayed to subjects in real-time (visual feedback) was given as follows:

$$\tau_{fb}=\tau_{act}+G\times sign\left(\tau_{act}-\tau_{d}\right){\left(\tau_{act}-\tau_{d}\right)}^{2},$$ (11)

where *τ*_*act*_ denotes the actual total elbow torque produced by subjects; *G*, the feedback gain; and *τ*_*d*_, the target total torque. The feedback gain *G* controls the sensitivity to an error; thus, a higher value of *G* requires more accurate target tracking. Subjects performed 60 trials, including 12 pre-test and 12 post-test trials. The middle 36 trials were the learning phase, which consisted of six blocks (L1–L6) × six trials. During pre- and post-tests, the feedback gain was 0.02. During the learning phase, the *G*-value was adjusted based on the lowest duration of time from the margin *t*_out_ observed at the pre-test as follows: if the lowest *t*_*out*_ ≤ 0.5 s, *G* = 0.025; for 0.5 s < *t*_*out*_ ≤ 1.0 s, *G* = 0.02; for 1.0 s < *t*_*out*_ ≤ 5.0 s, *G* = 0.015, and for 5.0 s < *t*_*out*_, *G* = 0.01; the better the performance in pre-test, the higher the *G*-value was set to ensure the task was difficult. After each block of six trials, if at least one of the *t*_*out*_ scores was 10% lower than the best *t*_*out*_ score recorded in the previous block, the value for *G* was increased by 0.005. After one tracking trial, the value of *t*_*out*_ was presented to the subjects as feedback information. Subjects were instructed to reduce the *t*_*out*_, i.e. reduce the duration of time outside the margin. In all trials, subjects took a short break when they felt fatigue.

#### Data analysis

All recorded data in all tasks were down-sampled with a 10-point average. EMG data were full-wave rectified, filtered using a second-order Butterworth low-pass filter with a 3-Hz cut-off frequency, and normalized to the value during maximum voluntary contraction. We considered the filtered EMG data as representative of the muscle tension.

In the target hand force production task, 48 data points of mean value and standard deviation of EMG (16 directions × 3 magnitudes of target force) were used to determine the coefficient of variation by linear regression. The determined coefficient of variation was used for the simulation experiments and calculation of the normalized index of synergy for the target total torque production task and the tracking-ability learning task.

Using the mean values of EMG and elbow torque for 1.5 s in the target total torque production task, the measured index of synergy and the normalized index of synergy were calculated. The variation in EMG of each muscle across 50 trials of the task was divided into the UCM component that has no effect on the total elbow torque and the ORT component that does have an effect. The Fisher Z-transformed value of the difference between the UCM and ORT components normalized by the total variance (Eq (10)) was defined as the index of synergy. Using the coefficient of variation in each muscle, the measured variance of the EMG of each muscle was transformed so that it was distributed according to the signal-independent mixture of Gaussian distributions (Eq (4)). Then, the UCM analysis was applied for the converted variance of EMG to calculate the normalized index of synergy.

In the tracking-ability learning task, the time series of the EMG and elbow torque data in standard half-sine waves were used for analysis because target amplitude and period were invariant across trials. We used the mean values of EMG and elbow torque ± 0.25 s from the peak of a target. Because the target trajectory in one trial contained four standard half-sine waves corresponding to the two flexion and two extension movements, the pre- and post-tests contained 24 data series of flexion and extension movements (12 trials × two flexion or two extension half-sine waves). From the variance across these 24 data series, the measured and normalized indices of synergy were calculated similar to the target total torque production task. Moreover, we calculated the ratio of the change in the normalized index of synergy from the pre-test to post-test with that of the measured index of synergy. We confirmed the same tendency of results of the UCM analysis in both elbow flexion and extension movements; thus, results in elbow flexion movements are shown.

#### Statistical analysis

In both the simulation experiments and the target total torque production task, we evaluated the difference between the UCM component and ORT component for measured and normalized muscle tensions. We performed a two-way repeated measures analysis of variance (ANOVA) to examine the effect of component variance (UCM and ORT components) and variance transformation (measured and normalized) with respect to the value of component variance. We performed a one-sample *t*-test (*α* = 0.05) to examine the difference between the index of synergy and the value of no-coordination, where the UCM component is equal to the ORT component. A paired *t*-test (*α* = 0.05) was also performed to examine the difference between the right and left elbow torques in the measurement experiments.

In the tracking-ability learning task, we evaluated the change in the tracking performance, UCN component, ORT component, and the index of synergy through learning. We performed a one-way ANOVA to examine the effect of learning with respect to the duration of time out of margin (*t*_*out*_) and the feedback gain (*G*). The Bonferroni method was used for post-hoc tests (*α* = 0.05). A two-way ANOVA was performed to examine the effect of learning (pre- and post-tests) and variance transformation (measured and normalized) with respect to the UCM component, the ORT component, and the index of synergy. We performed a one-sample *t*-test (*α* = 0.05) to examine the difference between the index of synergy and the value of no coordination, and whether the ratio of change in the normalized index of synergy to the measured index of synergy was above chance level (50%).

## Results

### Simulation Experiments

**Fig 5:**
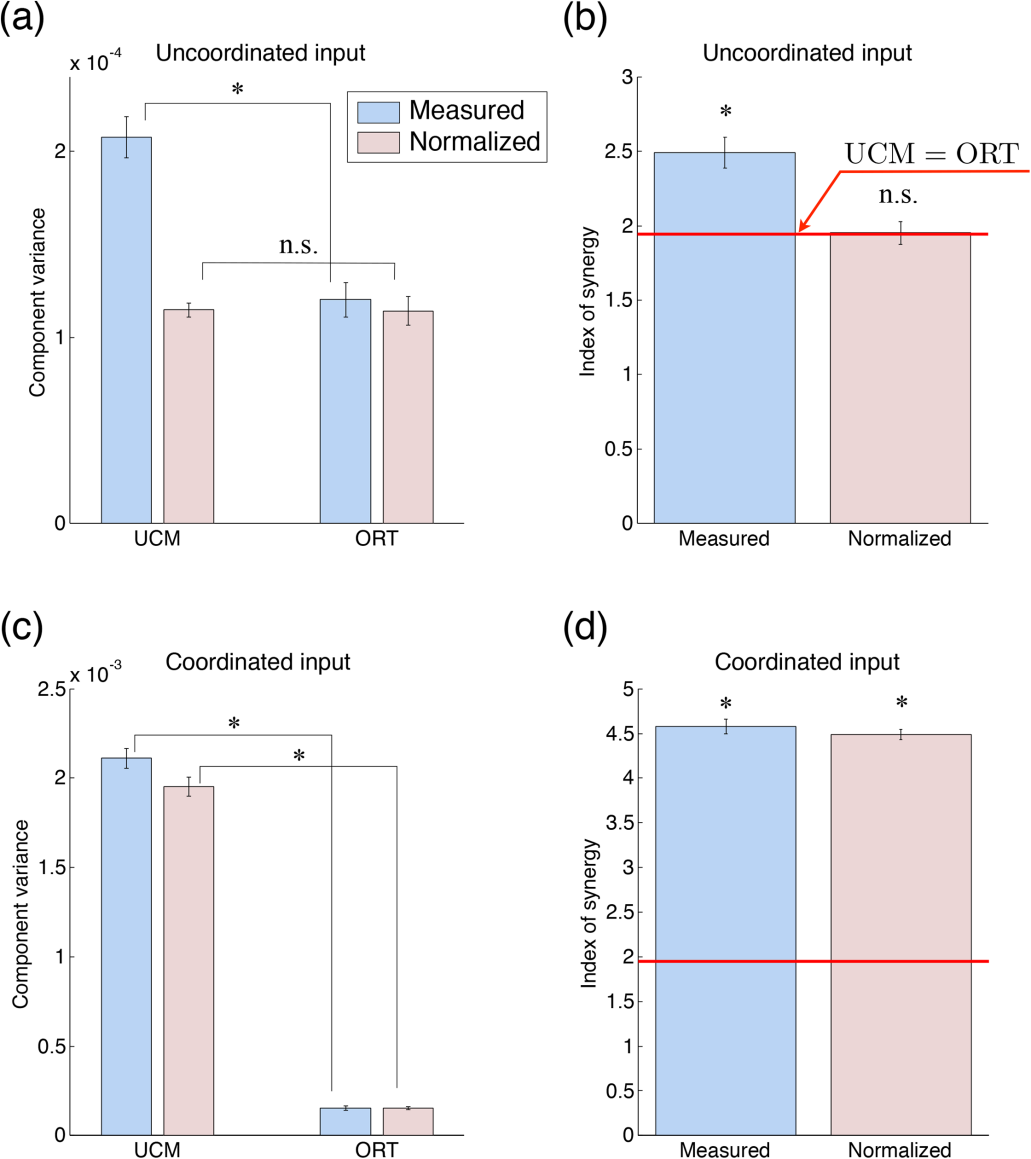
Results of the UCM analysis in simulation experiments. The UCM and ORT components for the uncoordinated input (a) and those for the coordinated input (c). The blue and red bars denote the component variance for the measured muscle tensions *T*_*i*_ and that for the normalized muscle tensions ${\hat{T}}_{n}$, respectively. The index of synergy for the uncoordinated input (b) and that for the coordinated input (d). The red line indicates the value of no coordination (where the UCM component is equal to the ORT component). The asterisk indicates significant difference (*P* < 0.05) between the UCM and ORT components ((a) and (c)) and from the red line ((b) and (d)), and n.s. indicates a non-significant difference. Specific values are listed in S2 Table.

We conducted simulation experiments to demonstrate that the properties of SDN form the coordination of muscle tensions, and the validity of our proposed method. Fig 5 shows the results of UCM analysis in the simulation experiments. Fig 5A and 5C show the UCM and ORT components for each input, and for the measured and normalized muscle tensions. Fig 5B and 5D show the measured and normalized indices of synergy for each input.

For uncoordinated input, a two-way ANOVA indicated a significant difference between the UCM and ORT components (*F*(1, 36) = 273.1, *P* = 2.18 × 10^−18^ < 0.05) and a significant interaction (*F*(1, 36) = 267.2, *P* = 3.08 × 10^−18^ < 0.05), suggesting that our proposed method affects the ratio of the UCM and ORT components (Fig 5A). The measured UCM component was significantly larger than the measured ORT component (*t*(9) = 17.5, *P* = 2.87 × 10^−8^ < 0.05), while the normalized UCM component was not significantly different from the normalized ORT component (*t*(9) = 0.17, *P* = 0.87). Also, the measured index of synergy was significantly higher than the value for no coordination (*t*(9) = 16.6, *P* = 4.68 × 10^−8^ < 0.05), while the normalized index of synergy was not significantly different (*t*(9) = 0.23, *P* = 0.82, Fig 5B). The significant difference between the measured UCM and ORT components indicates that the properties of the SDN caused the coordinated structure of the muscle tensions, despite the uncoordinated input. Moreover, the difference was eliminated in the normalized UCM and ORT components, which indicates that our proposed method could remove the effects of SDN on the variance structure.

For coordinated input, two-way ANOVA indicated a significant difference between the UCM and ORT components (*F*(1, 36) = 22360, *P* = 6.78 × 10^−52^ < 0.05), and a significant interaction (*F*(1, 36) = 40.4, *P* = 2.33 × 10^−7^ < 0.05, Fig 5C). In contrast to the results for uncoordinated input, both the measured and normalized UCM components were significantly larger than the measured and normalized ORT components, respectively (measured: *t*(9) = 107.8, *P* = 2.58 × 10^−15^ < 0.05; normalized: *t*(9) = 110.4, *P* = 2.08 × 10^−15^ < 0.05). Furthermore, both the measured and normalized indices of synergy were significantly higher than the values for no coordination (measured: *t*(9) = 102.3, *P* = 4.12 × 10^−15^ < 0.05; normalized: *t*(9) = 136.4, *P* = 3.10 × 10^−16^ < 0.05, Fig 5D). These results indicate that even if the inputs were coordinated, the normalized index of synergy continued to show coordinated structure.

The results of the simulation experiments demonstrated that the properties of SDN generated a coordinated structure of muscle tensions independent of the coordination of inputs. Moreover, it was also shown that our proposed method, the normalized index of synergy, could appropriately represent the coordinated structure of motor commands.

### Target Hand Force Production Task

**Fig 6:**
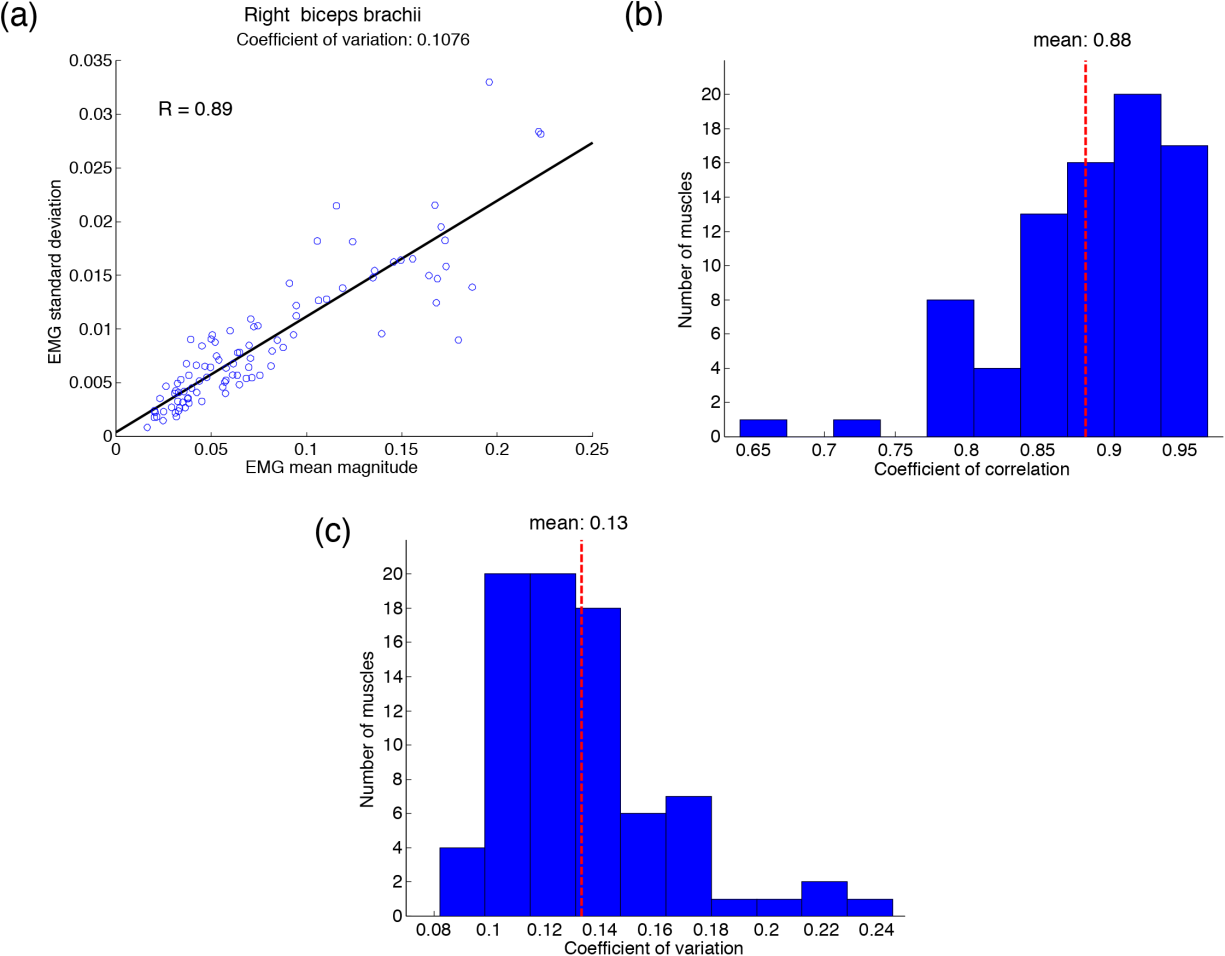
Determined coefficient of variation of each muscle. (a) A relationship between the mean magnitude and standard deviation of the EMG of one muscle of a typical subject. The slope indicates the coefficient of variation. The histogram of coefficient of correlation (b) and coefficient of variation (c) of all muscles of all subjects. The red dashed-line denotes the mean value.

The target hand force production task was conducted to determine the coefficient of variation of each muscle. Fig 6A shows one example of the properties of the SDN in the right biceps brachii of a typical subject. The standard deviation of EMG increased linearly with the mean magnitude of EMG, and the value of the correlation coefficient (*R* = 0.89) indicated a strong correlation. The histogram of correlation coefficients of all muscles in all subjects is shown in Fig 6B, where all muscles in all subjects showed properties of SDN (mean *R* = 0.88 ± 0.06). The linear regression slope represents the coefficient of variation of each muscle. Fig 6C shows the histogram of all coefficients of variation in all subjects. The value of the coefficient of variation varied from 0.082 to 0.246 (mean 0.13 ± 0.03).

### Target Total Torque Production Task

**Fig 7:**
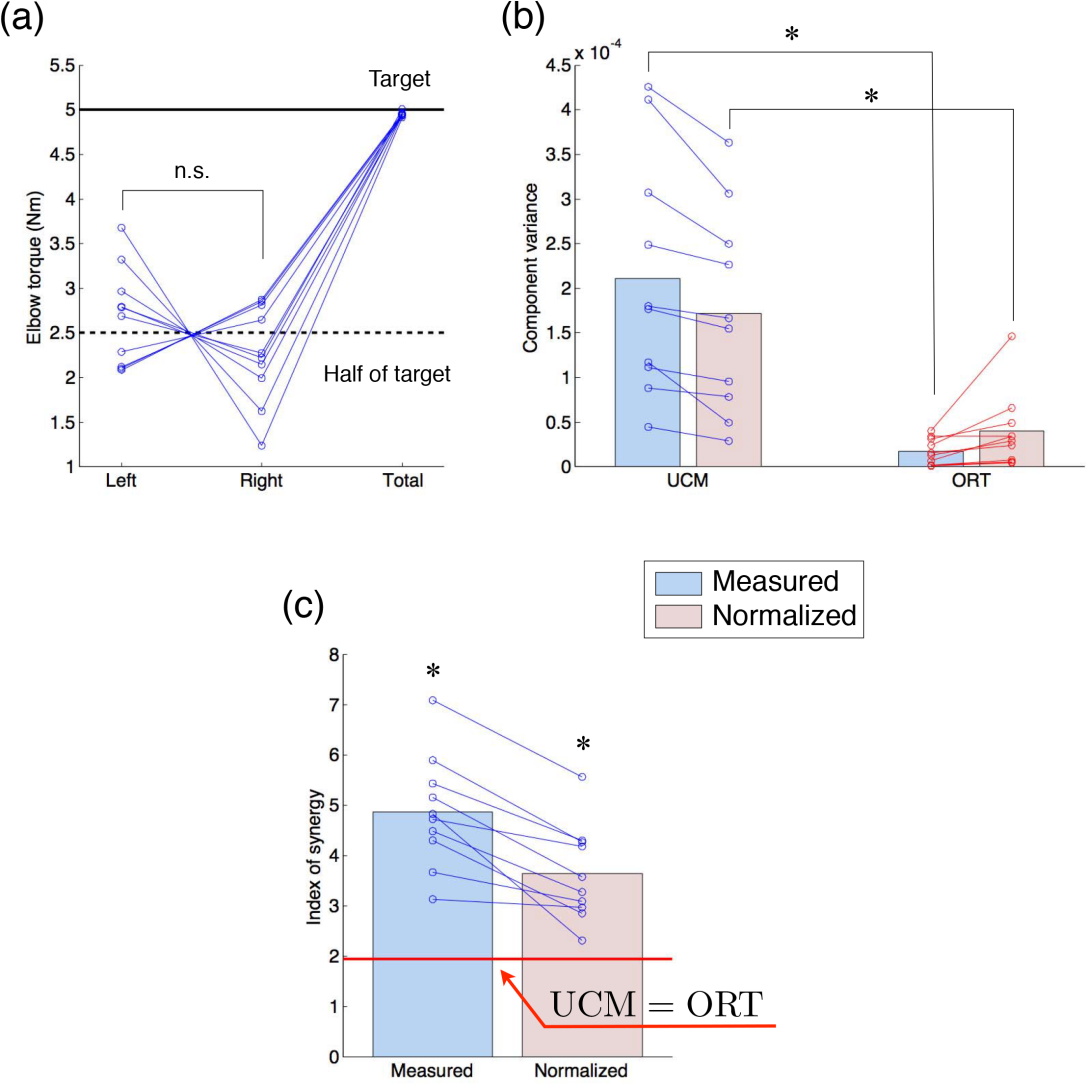
Results of the measurement experiments of the target total torque production task. (a) The blue circle indicates individual left, right and total elbow torques of all subjects. (b) The UCM component and the ORT component of all subjects. (c) The index of synergy of all subjects. The asterisk indicates a significant difference (*P* < 0.05), and n.s. indicates a non-significant difference.

In the target total torque production task, subjects performed the same task as in the simulation experiment to evaluate the coordination of motor commands. Fig 7A indicates the left, right, and total elbow torques produced by all subjects. All subjects could accurately produce target total torque (5 N·m). Although all subjects were right-handed, a significant bias between right and left elbow torques was not observed (*t*(9) = −1.20, *P* = 0.26), suggesting little effect of hand dominance on the task performance. Fig 7B shows both measured and normalized UCM and ORT components. Two-way ANOVA indicated a significant difference between the UCM and ORT components (*F*(1, 36) = 32.7, *P* = 1.65 × 10^−6^ < 0.05), without a significant interaction (*F*(1, 36) = 1.2, *P* = 0.28), and both the measured and normalized UCM components were significantly larger than the measured and normalized ORT components, respectively (measured: *t*(9) = 4.83, *P* = 9.37 × 10^−4^ < 0.05; normalized: *t*(9) = 4.67, *P* = 1.20 × 10^−3^ < 0.05). Then, the measured and normalized indices of synergy were significantly higher than the values of no coordination (measured: *t*(9) = 8.27, *P* = 1.70 × 10^−5^ < 0.05; normalized: *t*(9) = 5.66, *P* = 3.10 × 10^−4^ < 0.05, see UCM = ORT line) as shown in Fig 7C. These results demonstrated that the coordination of muscle tensions was formed during the input step to each muscle, i.e. by the motor commands. Therefore, these results show that the subjects coordinated motor commands to produce the target total elbow torque.

### Results of Tracking-Ability Learning Task

**Fig 8:**
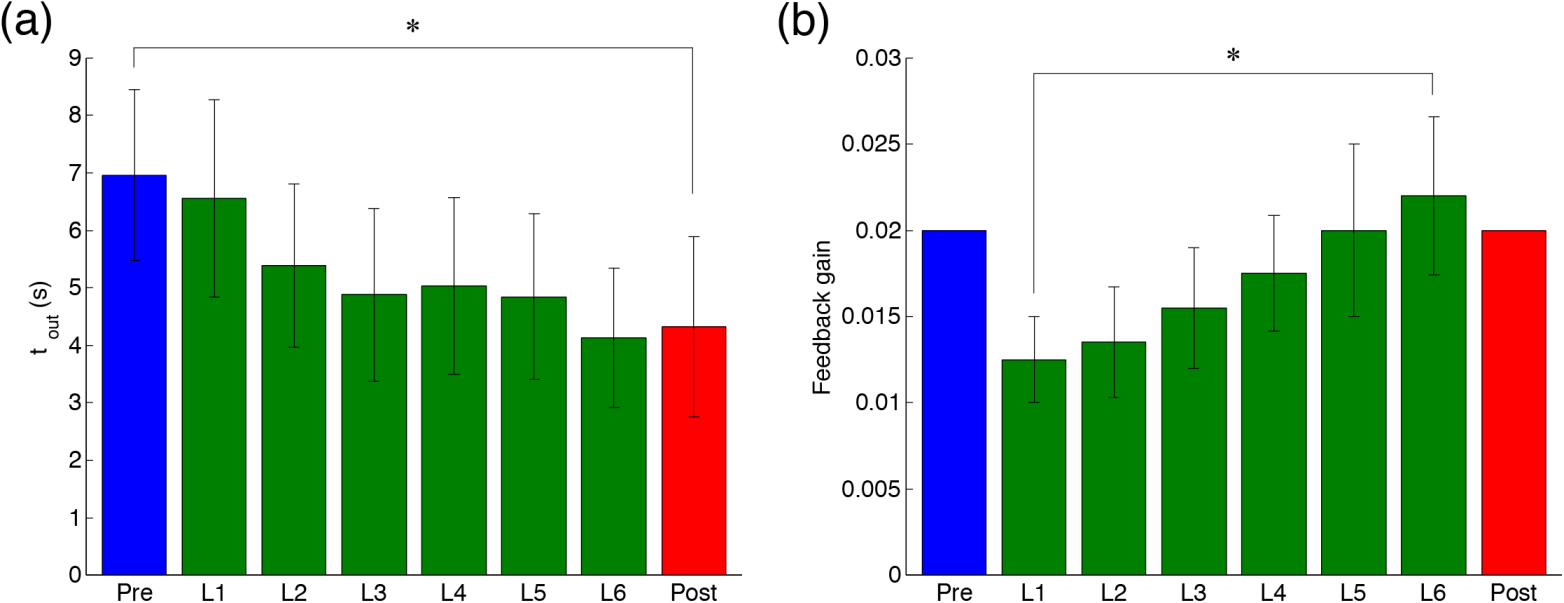
Change in the tracking performance through motor learning. (a) The duration of out of margin *t*_*out*_. (b) The feedback gain *G* (Eq (11)). The blue, green and red bars indicate the pre-test, the learning phases, and the post-test, respectively. The asterisk indicates a significant difference (*P* < 0.05). Specific values are listed in S3 Table.

To demonstrate the usability of our proposed method, we conducted a tracking-ability learning task. Fig 8 shows the tracking performance, the duration of time outside the margin *t*_*out*_ (Fig 8A) and the feedback gain *G* (in Eq (11) and Fig 8B), for all subjects. The duration outside the margin *t*_*out*_ gradually decreased through learning, and was significantly smaller in post-test than in pre-test (*F*(7, 72) = 4.1, *P* = 7.74 × 10^−4^ < 0.05). This result indicates that subjects could significantly learn to accurately track the target trajectory of total elbow torque. The feedback gain *G* gradually increased through learning, and was significantly larger in L6 (sixth learning phase) than in L1 (*F*(7, 72) = 10.0, *P* = 1.10 × 10^−8^ < 0.05). This result indicates that subjects could gradually learn a more difficult task in later learning phases.

**Fig 9:**
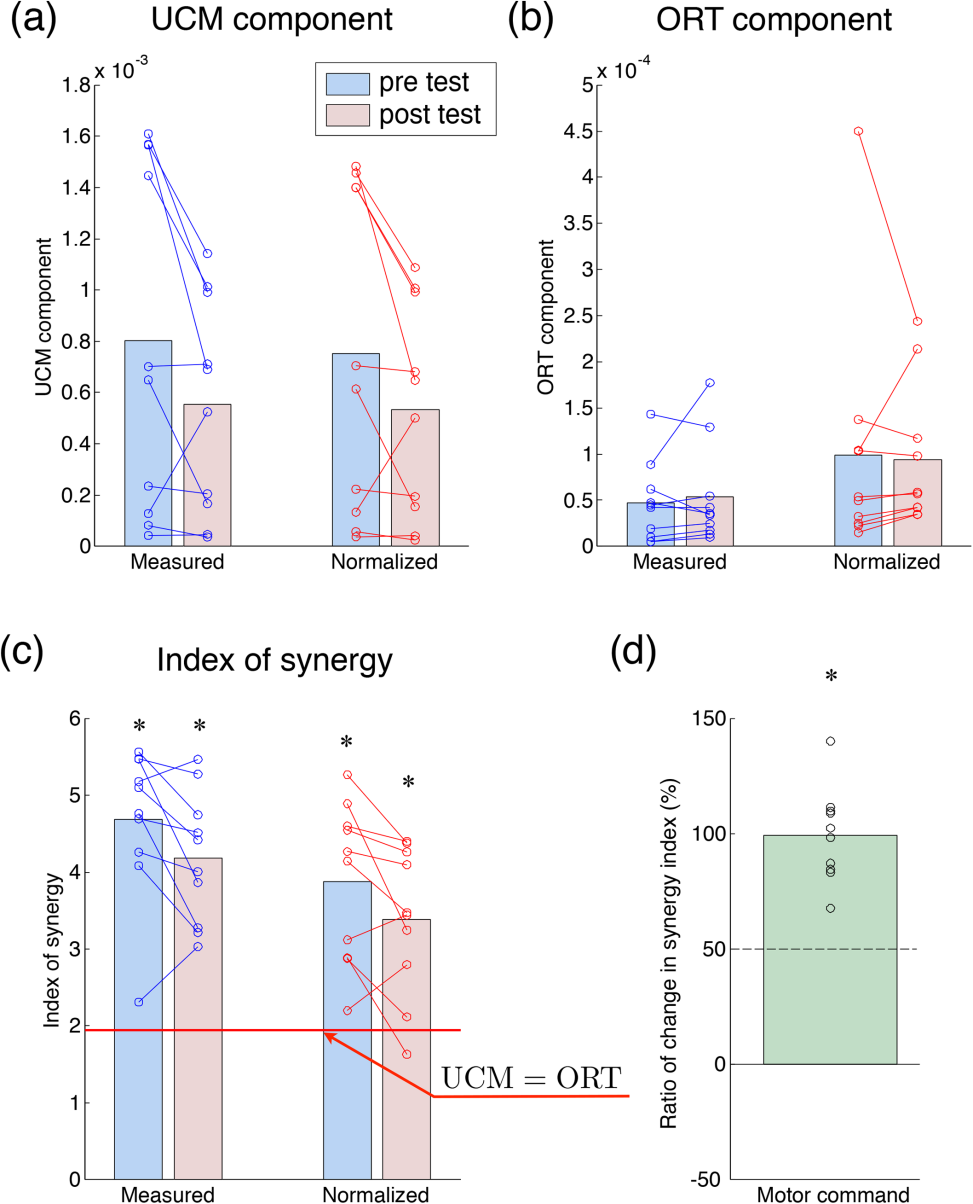
Results of the UCM analysis in tracking-ability learning task. The UCM component (a), the ORT component (b), and the index of synergy (c) pre- and post-test. (d) The ratio of the change in the coordination index of synergy of the motor commands to that in the index of synergy of muscle tensions. The circle indicates each subject. The asterisk denotes a significant difference (*P* < 0.05).

Fig 9 shows the results of the UCM analysis. Two-way ANOVA indicated that the UCM and ORT components and index of synergy did not significantly change through learning (UCM: *F*(1, 36) = 1.82, *P* = 0.19 in Fig 9A; ORT: *F*(1, 36) = 1.40 × 10^−3^, *P* = 0.97 in Fig 9B; and Index: *F*(1, 36) = 2.74, *P* = 0.11 in Fig 9C). However, the UCM component showed a tendency to decrease (Fig 9A), and thus the index of synergy also showed a tendency to decrease (Fig 9C). The indices of synergy for all conditions were significantly higher than the values of no coordination (pre and measured: *t*(9) = 8.87, *P* = 9.59 × 10^−6^ < 0.05; pre and normalized: *t*(9) = 5.95, *P* = 2.17 × 10^−4^ < 0.05; post and measured: *t*(9) = 8.29, *P* = 1.66 × 10^−5^ < 0.05; post and normalized: *t*(9) = 4.72, *P* = 1.10 × 10^−3^ < 0.05, in Fig 9C). These results indicate that the synergy for stabilizing the total elbow torque was weakened through motor learning, but remained higher than the value of no coordination before and after learning. Fig 9D shows the ratio of change in the normalized index of synergy to that in the measured index of synergy. The greatest amount of change in the index of synergy of muscle tensions was dominated by the change in the index of synergy of motor commands (mean 99.4 ± 20.1%, which significantly higher than the chance level, *t*(9) = 7.75, *P* = 2.85 × 10^−5^ < 0.05). This result indicates that the change in the synergy of the motor commands accounted for the greater part of that of the muscle tensions through short-term learning.

## Discussion

The present study proposed a method to evaluate the coordination of motor commands by eliminating the effects of the musculoskeletal system from the measured coordination of motor outputs. We investigated the effects of SDN in the muscle on the measured coordination. In the simulation experiments, we demonstrated that the differences of the degrees of variability among muscle tensions owing to the SDN produced the coordination of motor outputs, i.e. muscle tensions (Fig 5A and 5B). Then, the normalized index of synergy was calculated by the transformation from signal-dependent distributions of an estimated mixture of Gaussian distributions to signal-independent distributions and a UCM analysis. The normalized index of synergy could appropriately estimate the coordination of inputs from the motor outputs by eliminating the effect of the SDN in the simulation experiments (Fig 5). Therefore, we showed that our proposed method could evaluate the coordination of motor commands by removing the effects of SDN. Moreover, we applied our proposed method to the total torque production task performed by human subjects. The normalized index of synergy indicated that the subjects coordinated their motor commands to stabilize the total elbow torque (Fig 7). Finally, in the tracking-ability learning task, we showed that the change in the measured coordination of muscle tensions through motor learning was dominated by the change in the coordination of motor commands (Fig 9D). These results demonstrate the utility of our proposed method.

### Model Assumptions

The normalized index of synergy depends on certain assumptions. The first is that the standard deviation of the signal-independent Gaussian distribution (*β* in Eq (3)) is invariant across all inputs. The signal-independent Gaussian distribution is the distribution of muscle tensions without an effect of the SDN (left side of Fig 2B), which is considered as the motor command in this study. In the framework of UCM analysis, uncoordinated distribution of motor elements shows invariant variability across all elements [4]. When the input to each muscle is unique and uncoordinated, the estimated motor command is required to show the invariant variability across all commands based on the UCM framework. Therefore, the invariant standard deviation across all inputs was assumed so that the motor command with above property of distribution could be calculated using the UCM framework. Previous studies using UCM analysis examined the coordinated structure of motor outputs affected by SDN, but did not attempt to eliminate the effect of SDN. In this study, we proposed the model with invariant variability across all inputs as the first step in our UCM analysis at the motor-command level. Assessment of the validity of the assumption of the invariant standard deviation across all inputs will be examined in future studies. The second assumption is that the variability of the muscle tension varies according to the SDN. Many previous studies have reported that the distribution of muscle tension shows the property of the SDN [5–7]. A previous simulation study revealed that the linear scaling of SDN is a natural byproduct of the physiological organization of the motor-unit pool [6]. Thus, the assumption of the muscle tension with SDN was based on the physiological properties of muscle.

### Simulation Experiments for Other Types of Uncoordinated Input

The results of the simulation experiments showed the coordination of muscle tensions, as shown in Fig 5A and 5B, when the unique combination of the inputs shown in Table 1 was given. This result can be interpreted as showing that the muscle tensions were coordinated because of the properties of SDN despite the uncoordinated input. The coordination of muscle tensions in the framework of UCM analysis was the result of the shape of the distribution of muscle tensions. Because the degrees of variability of muscle tensions were different among muscles, the shape of the output of muscle tensions would be along the UCM. However, the uncoordinated input does not always generate coordinated muscle tensions. As shown in S1 Fig, for instance, when uncoordinated inputs (S1 Table) were given to the musculoskeletal system of Eq (1), the distribution of muscle tensions showed a significantly higher ORT component than the UCM component (*t*(9) = −32.1, *P* = 1.36 × 10^−10^ < 0.05), in contrast to those in Fig 5. The index of synergy of muscle tensions was significantly lower than the value of no coordination (*t*(9) = −54.0, *P* = 1.28 × 10^−12^ < 0.05), which indicates a negative synergy that destabilizes the performance variable [3]. In earlier studies, a negative synergy has been observed when a quick change of the performance variable is required [23–25] and when the trial-by-trial adjustment of the performance variable is needed [26–27]. The results shown in S1 Fig indicate that the properties of SDN could also cause a negative synergy. Because the input was unique and not coordinated, such a negative synergy did not depend on the properties of the input. Applying our proposed method to the distribution of muscle tensions that shows a negative synergy in S1 Fig, the normalized UCM and ORT components were not significantly different (*t*(9) = 0.21, *P* = 0.84) and the normalized index of synergy was not significantly different from the value of no coordination (*t*(9) = 0.34, *P* = 0.74). Thus, our proposed method can remove the effect of SDN in producing not only the synergy, as shown in Fig 5, but also the negative synergy, as shown in S1 Fig

### Mechanism of Coordination in the Target Total Torque Production Task

In the target total torque production task, the subjects showed significantly high values of the normalized index of synergy (Fig 7). This result suggests that the subjects coordinated their motor commands to stably produce the target total elbow torque. In this study, the motor command was the muscle tension without the effect of SDN, which is considered as the input commands to each muscle in the planning stage or as the input signals to the motor pool. Recently, a low-dimensional control variable between the body and the motor pool, the muscle synergies [28–30] (or the muscle modes in the framework of UCM [31–33]), has been studied. However, because such a low-dimensional control variable is hypothetical [34–36], the mechanism that forms the coordination of motor commands remains unclear. In our future work, we will test whether the transformed motor commands (eliminating the effect of the SDN) are decodable from the brain activity or connectivity to examine the neural mechanism forming the coordination of the motor command, i.e. the neural basis of the synergy.

### Change in Synergy through Motor Learning

To demonstrate the utility of our proposed method, we calculated the normalized index of synergy in the motor learning task, wherein the subjects learned to track the target trajectory of their total elbow torque. The subjects repeatedly tracked a randomly generated target trajectory and could improve their tracking ability (Fig 8). With the improvement of tracking ability, the UCM component decreased and the measured and normalized indices of synergy dropped (Fig 9A, 9B and 9C). Although the tracking ability was significantly improved with learning, the ORT component did not change. The improvement of tracking accuracy should lead to the decrease of the ORT component. The reason that the ORT component did not change is that the tracking ability was evaluated across the whole movement, whereas the ORT component contains data only from around the peak of the standard half-sine waves of the target. The index of synergy decreased because of the decrease in the UCM component and the unchanged ORT component, which means that the total variability (UCM + ORT components) decreased. Therefore, the subjects seemed to adopt a strategy in which they decreased the total variability of their muscle tensions. In contrast, earlier studies that used the same motor learning task as our study reported that the UCM component increased, following which the index of synergy increased [21–22]. These earlier studies used two fingers (the index and middle fingers) to produce the total force to track the target trajectory. Another earlier study reported that the amplitude of variability of joint torque in distal parts was larger than that in proximal parts [8]. In earlier studies, because the amplitude of variability of the finger force is larger than that of the elbow, it would be difficult to adopt a strategy in which the variability of finger force was reduced through learning. Thus, the subjects in earlier studies adopted a strategy in which the variability of finger force was distributed more along a direction that did not affect the performance variable, i.e. in which the synergy was enhanced. Similarly, it was reported that the subjects increased the UCM component and enhanced the synergy when the reduction of the variability of motor elements was difficult because of the task conditions [37–38]. By contrast, because the reduction of the variability of elbow joint torque (and of the related muscle tensions) was possible in this study, the subjects adopted a strategy in which the total variability of motor elements was reduced. Similarly, the decrease of the UCM component through motor learning has previously been reported in simple motor tasks, e.g. the pointing task [39–40], and the multi-finger force production task (not trajectory-tracking task) [41–42]. Combining the earlier reports and the results of this study, we suggest that humans adopt a strategy in which the synergy is enhanced when the decrease of the variability of motor elements is difficult, or adopt a strategy in which the synergy is reduced when the decrease of the variability of motor elements is easy.

### Effect of SDN on Kinematic Coordination

In this study, we focused on the coordination of muscle tensions to produce isometric elbow torque to simplify the problem, and proposed a method to normalize the variability of muscle tensions to remove the effect of SDN. In coordinated movements, the coordination among the kinematic motor elements, e.g. the joint angles and the angular velocity, are also important. Therefore, we need to extend this method to problems involving kinematic motor elements in future studies. However, the variability of kinematic motor elements does not show a direct effect of the SDN of muscles. The human joint has a spring-like property that reflects the equilibrium of the agonist and antagonist muscles [43]. The decrease in variability of the joint and hand kinematics resulting from adjustments in the joint stiffness, which corresponds to the spring constant of the joint, has been reported in simulation [44], and measurement experiments [45–46]. Thus, for the normalization of variability of kinematics, we must consider that joint stiffness can affect the variability of kinematics. When extending the method to a task with kinematic motor elements, we hope that we can examine the role of coordination depending on the motor commands and on the properties of the musculoskeletal system in various other motor tasks.

## Appendix

### A: Derivation of the Equation of Transformation from the Muscle Tension to the Motor Command

In a signal-dependent mixture of Gaussians distribution that is estimated by a maximum-likelihood estimation using an EM algorithm, the mean and standard deviation of the *k-*th Gaussian distribution are *μ*_*k*_ and *α*_*i*_ *μ*_*k*_, respectively. Using the measured EMG data *T*_*m*_ and a responsibility *γ*_*nk*_, the variance of the *k*-th distribution is represented as follows:

$${\left(\alpha \mu_{k}\right)}^{2}=\frac{1}{N_{k}}\sum_{n}\gamma_{nk}{\left(T_{n}-\mu_{k}\right)}^{2},$$ (A1)

where $N_{k}=\sum_{n}\gamma_{nk}$, and *n* denotes the number of measured EMG data points. An amount of change *r*_*nk*_ was added to the measured EMG data *T*_*n*_ so that the changed standard deviation of the *k*-th distribution is *β*. Then, the variance *β* ^2^ is represented as follows:

$$\beta^{2}=\frac{1}{N_{k}}\sum_{n}\gamma_{nk}{\left(T_{n}+r_{nk}-\mu_{k}\right)}^{2}.$$ (A2)

Using Eqs (A1) and (A2), the amount of change *r*_*nk*_ is represented as follows:

1(αμk)2Nk∑nγnk(Tn−μk)2=1β2Nk∑nγnk(Tn+rnk−μk)2(Tn−μk)2(αμk)2=(Tn+rnk−μk)2β2rnk=(Tn−μk)(βαμk−1). (A3)

Because the measured EMG data *T*_*n*_ is affected by *γ*_*nk*_ from the *k*-th Gaussian distribution, the transformed data ${\hat{T}}_{n}$ is as follows:

$${\hat{T}}_{n}=T_{n}+\sum_{k}\gamma_{nk}r_{nk}.$$ (A4)

Using Eqs (A3) and (A4), we can derive Eq (4).

## Supporting Information

S1 FigResults of the UCM analysis for the other uncoordinated input in the simulation experiments.The UCM component and the ORT component for the other uncoordinated input as shown in S1 Table (a). The blue and red bars denote the component variance for the measured muscle tensions *T*_*i*_ and that for the normalized muscle tensions ${\hat{T}}_{n}$, respectively. The index of synergy for the other uncoordinated input (b). The red line indicates the value of no-coordination (the UCM component is equal to the ORT component). The asterisk indicates a significant difference (*P* < 0.05), and n.s. indicates a non-significant difference. Specific values are listed in S4 Table.(EPS)

S1 Table Uncoordinated input generating negative synergy in the simulation experiments. (DOCX)

S2 Table Specific values of the results of UCM analysis in simulation experiment shown in Fig 5. (DOCX)

S3 Table Specific values of the change in the tracking performance shown in Fig 8. (DOCX)

S4 Table Specific values of results of UCM analysis in simulation experiment shown in S1 Fig. (DOCX)
