# Supplementary material for: Normalized Index of Synergy for Evaluating the Coordination of Motor Commands
Source: PLoS One. 2015 Oct 16;10(10):e0140836. doi: 10.1371/journal.pone.0140836 (PMC4608756; doi:10.1371/journal.pone.0140836)
Supplement: S1 Table — (DOCX) [file pone.0140836.s002.docx]

|  | $e_{1}$ | $e_{2}$ | $e_{3}$ | $e_{4}$ | $e_{5}$ | $e_{6}$ | $e_{7}$ | $e_{8}$ |
| --- | --- | --- | --- | --- | --- | --- | --- | --- |
| Uncoordinated input | 0.02 | 0.01 | 0.05 | 0.01 | 0.02 | 0.01 | 0.06 | 0.01 |
