# Supplementary material for: Normalized Index of Synergy for Evaluating the Coordination of Motor Commands
Source: PLoS One. 2015 Oct 16;10(10):e0140836. doi: 10.1371/journal.pone.0140836 (PMC4608756; doi:10.1371/journal.pone.0140836)
Supplement: S2 Table — (DOCX) [file pone.0140836.s003.docx]

Fig. 5a

|  | Measured UCM | Normalized UCM | Measured ORT | Normalized ORT |
| --- | --- | --- | --- | --- |
| Session 1 | $2.15\times{10}^{-4}$ | $1.13\times{10}^{-4}$ | $1.13\times{10}^{-4}$ | $1.09\times{10}^{-4}$ |
| Session 2 | $1.91\times{10}^{-4}$ | $1.14\times{10}^{-4}$ | $1.21\times{10}^{-4}$ | $1.17\times{10}^{-4}$ |
| Session 3 | $2.15\times{10}^{-4}$ | $1.20\times{10}^{-4}$ | $1.26\times{10}^{-4}$ | $1.19\times{10}^{-4}$ |
| Session 4 | $2.09\times{10}^{-4}$ | $1.14\times{10}^{-4}$ | $9.97\times{10}^{-5}$ | $1.02\times{10}^{-4}$ |
| Session 5 | $2.06\times{10}^{-4}$ | $1.10\times{10}^{-4}$ | $1.18\times{10}^{-4}$ | $1.10\times{10}^{-4}$ |
| Session 6 | $1.99\times{10}^{-4}$ | $1.17\times{10}^{-4}$ | $1.21\times{10}^{-4}$ | $1.08\times{10}^{-4}$ |
| Session 7 | $2.02\times{10}^{-4}$ | $1.09\times{10}^{-4}$ | $1.28\times{10}^{-4}$ | $1.23\times{10}^{-4}$ |
| Session 8 | $1.98\times{10}^{-4}$ | $1.13\times{10}^{-4}$ | $1.34\times{10}^{-4}$ | $1.26\times{10}^{-4}$ |
| Session 9 | $2.09\times{10}^{-4}$ | $1.17\times{10}^{-4}$ | $1.17\times{10}^{-4}$ | $1.13\times{10}^{-4}$ |
| Session 10 | $2.30\times{10}^{-4}$ | $1.19\times{10}^{-4}$ | $1.24\times{10}^{-4}$ | $1.15\times{10}^{-4}$ |

Fig. 5b

|  | Measured | Normalized |
| --- | --- | --- |
| Session 1 | 2.59 | 1.99 |
| Session 2 | 2.40 | 1.93 |
| Session 3 | 2.48 | 1.95 |
| Session 4 | 2.68 | 2.06 |
| Session 5 | 2.50 | 1.94 |
| Session 6 | 2.44 | 2.03 |
| Session 7 | 2.40 | 1.83 |
| Session 8 | 2.33 | 1.83 |
| Session 9 | 2.53 | 1.98 |
| Session 10 | 2.57 | 1.98 |

Fig. 5c

|  | Measured UCM | Normalized UCM | Measured ORT | Normalized ORT |
| --- | --- | --- | --- | --- |
| Session 1 | $2.14\times{10}^{-3}$ | $1.98\times{10}^{-3}$ | $1.63\times{10}^{-4}$ | $1.49\times{10}^{-4}$ |
| Session 2 | $2.09\times{10}^{-3}$ | $1.94\times{10}^{-3}$ | $1.44\times{10}^{-4}$ | $1.49\times{10}^{-4}$ |
| Session 3 | $2.11\times{10}^{-3}$ | $1.95\times{10}^{-3}$ | $1.45\times{10}^{-4}$ | $1.46\times{10}^{-4}$ |
| Session 4 | $2.12\times{10}^{-3}$ | $1.95\times{10}^{-3}$ | $1.39\times{10}^{-4}$ | $1.45\times{10}^{-4}$ |
| Session 5 | $2.15\times{10}^{-3}$ | $1.99\times{10}^{-3}$ | $1.43\times{10}^{-4}$ | $1.50\times{10}^{-4}$ |
| Session 6 | $2.08\times{10}^{-3}$ | $1.93\times{10}^{-3}$ | $1.67\times{10}^{-4}$ | $1.63\times{10}^{-4}$ |
| Session 7 | $2.20\times{10}^{-3}$ | $2.05\times{10}^{-3}$ | $1.69\times{10}^{-4}$ | $1.78\times{10}^{-4}$ |
| Session 8 | $2.02\times{10}^{-3}$ | $1.87\times{10}^{-3}$ | $1.52\times{10}^{-4}$ | $1.52\times{10}^{-4}$ |
| Session 9 | $2.16\times{10}^{-3}$ | $2.00\times{10}^{-3}$ | $1.39\times{10}^{-4}$ | $1.48\times{10}^{-4}$ |
| Session 10 | $2.04\times{10}^{-3}$ | $1.88\times{10}^{-3}$ | $1.60\times{10}^{-4}$ | $1.55\times{10}^{-4}$ |

Fig. 5d

|  | Measured | Normalized |
| --- | --- | --- |
| Session 1 | 4.52 | 4.53 |
| Session 2 | 4.62 | 4.51 |
| Session 3 | 4.62 | 4.54 |
| Session 4 | 4.67 | 4.54 |
| Session 5 | 4.66 | 4.53 |
| Session 6 | 4.47 | 4.41 |
| Session 7 | 4.51 | 4.39 |
| Session 8 | 4.53 | 4.46 |
| Session 9 | 4.69 | 4.55 |
| Session 10 | 4.49 | 4.44 |
