# Supplementary material for: Normalized Index of Synergy for Evaluating the Coordination of Motor Commands
Source: PLoS One. 2015 Oct 16;10(10):e0140836. doi: 10.1371/journal.pone.0140836 (PMC4608756; doi:10.1371/journal.pone.0140836)
Supplement: S3 Table — (DOCX) [file pone.0140836.s004.docx]

Fig. 8a

|  | Pre | L1 | L2 | L3 | L4 | L5 | L6 | Post |
| --- | --- | --- | --- | --- | --- | --- | --- | --- |
| Sub. 1 | 4.46 | 3.86 | 2.83 | 2.69 | 3.01 | 2.68 | 2.68 | 2.23 |
| Sub. 2 | 5.97 | 4.59 | 3.73 | 4.29 | 3.27 | 4.75 | 2.68 | 2.56 |
| Sub. 3 | 6.36 | 6.51 | 5.06 | 2.79 | 3.54 | 4.83 | 3.18 | 3.63 |
| Sub. 4 | 9.15 | 8.29 | 6.35 | 6.00 | 5.84 | 6.01 | 3.85 | 5.14 |
| Sub. 5 | 5.01 | 4.61 | 4.39 | 3.92 | 4.42 | 3.50 | 3.01 | 2.87 |
| Sub. 6 | 6.75 | 8.08 | 7.12 | 4.31 | 6.94 | 7.68 | 5.99 | 7.67 |
| Sub. 7 | 7.18 | 8.79 | 5.27 | 7.35 | 5.57 | 3.89 | 4.56 | 4.15 |
| Sub. 8 | 8.26 | 7.77 | 6.09 | 5.62 | 5.37 | 3.76 | 5.22 | 4.41 |
| Sub. 9 | 7.48 | 5.38 | 5.34 | 4.96 | 3.91 | 4.81 | 4.12 | 4.62 |
| Sub. 10 | 8.95 | 7.71 | 7.67 | 6.87 | 7.38 | 6.54 | 5.99 | 5.97 |

Fig. 8b

|  | Pre | L1 | L2 | L3 | L4 | L5 | L6 | Post |
| --- | --- | --- | --- | --- | --- | --- | --- | --- |
| Sub. 1 | 0.02 | 0.015 | 0.02 | 0.02 | 0.025 | 0.03 | 0.03 | 0.02 |
| Sub. 2 | 0.02 | 0.015 | 0.015 | 0.015 | 0.015 | 0.015 | 0.02 | 0.02 |
| Sub. 3 | 0.02 | 0.015 | 0.015 | 0.015 | 0.02 | 0.02 | 0.02 | 0.02 |
| Sub. 4 | 0.02 | 0.01 | 0.01 | 0.015 | 0.015 | 0.015 | 0.02 | 0.02 |
| Sub. 5 | 0.02 | 0.015 | 0.015 | 0.02 | 0.02 | 0.025 | 0.025 | 0.02 |
| Sub. 6 | 0.02 | 0.01 | 0.01 | 0.01 | 0.015 | 0.015 | 0.02 | 0.02 |
| Sub. 7 | 0.02 | 0.015 | 0.015 | 0.02 | 0.02 | 0.025 | 0.03 | 0.02 |
| Sub. 8 | 0.02 | 0.01 | 0.01 | 0.015 | 0.015 | 0.015 | 0.015 | 0.02 |
| Sub. 9 | 0.02 | 0.01 | 0.015 | 0.015 | 0.015 | 0.02 | 0.02 | 0.02 |
| Sub. 10 | 0.02 | 0.01 | 0.01 | 0.01 | 0.015 | 0.015 | 0.02 | 0.02 |
