# Supplementary material for: Normalized Index of Synergy for Evaluating the Coordination of Motor Commands
Source: PLoS One. 2015 Oct 16;10(10):e0140836. doi: 10.1371/journal.pone.0140836 (PMC4608756; doi:10.1371/journal.pone.0140836)
Supplement: S4 Table — (DOCX) [file pone.0140836.s005.docx]

Fig. S1a

|  | Measured UCM | Normalized UCM | Measured ORT | Normalized ORT |
| --- | --- | --- | --- | --- |
| Session 1 | $1.04\times{10}^{-5}$ | $7.26\times{10}^{-6}$ | $2.22\times{10}^{-5}$ | $8.50\times{10}^{-6}$ |
| Session 2 | $1.05\times{10}^{-5}$ | $7.33\times{10}^{-6}$ | $2.02\times{10}^{-5}$ | $7.91\times{10}^{-6}$ |
| Session 3 | $1.11\times{10}^{-5}$ | $7.76\times{10}^{-6}$ | $2.06\times{10}^{-5}$ | $6.60\times{10}^{-6}$ |
| Session 4 | $0.92\times{10}^{-5}$ | $7.09\times{10}^{-6}$ | $1.80\times{10}^{-5}$ | $6.22\times{10}^{-6}$ |
| Session 5 | $1.10\times{10}^{-5}$ | $7.46\times{10}^{-6}$ | $2.07\times{10}^{-5}$ | $7.73\times{10}^{-6}$ |
| Session 6 | $1.10\times{10}^{-5}$ | $7.76\times{10}^{-6}$ | $2.20\times{10}^{-5}$ | $7.12\times{10}^{-6}$ |
| Session 7 | $1.13\times{10}^{-5}$ | $7.58\times{10}^{-6}$ | $2.21\times{10}^{-5}$ | $8.25\times{10}^{-6}$ |
| Session 8 | $1.18\times{10}^{-5}$ | $7.88\times{10}^{-6}$ | $2.38\times{10}^{-5}$ | $8.28\times{10}^{-6}$ |
| Session 9 | $1.11\times{10}^{-5}$ | $7.55\times{10}^{-6}$ | $2.16\times{10}^{-5}$ | $6.85\times{10}^{-6}$ |
| Session 10 | $1.05\times{10}^{-5}$ | $7.51\times{10}^{-6}$ | $2.08\times{10}^{-5}$ | $7.20\times{10}^{-6}$ |

Fig. S1b

|  | Measured | Normalized |
| --- | --- | --- |
| Session 1 | 1.19 | 1.79 |
| Session 2 | 1.29 | 1.87 |
| Session 3 | 1.33 | 2.11 |
| Session 4 | 1.28 | 2.08 |
| Session 5 | 1.32 | 1.91 |
| Session 6 | 1.25 | 2.03 |
| Session 7 | 1.28 | 1.86 |
| Session 8 | 1.25 | 1.90 |
| Session 9 | 1.27 | 2.04 |
| Session 10 | 1.26 | 1.99 |
